# Enhancing the lives of older refugees: an evaluation of a training resource

**DOI:** 10.1186/s13033-016-0067-5

**Published:** 2016-04-29

**Authors:** Shameran Slewa-Younan, Yvonne Santalucia, Regina McDonald, Marisa Salem

**Affiliations:** Centre for Health Research, School of Medicine, Western Sydney University, Campbelltown Campus, Sydney, Australia; Multicultural Health, South Western Sydney Local Health District, Liverpool Hospital Eastern Campus, Sydney, Australia; Specialist Mental Health Services for Older People, Braeside Hospital Hammond Care, South Western Sydney Local Health District, Sydney, Australia; NSW Refugee Health Service, South Western Sydney Local Health District, Liverpool, Sydney, Australia; Liverpool Hospital, South Western Sydney Local Health District, Locked Bag 7103, Liverpool BC, Sydney, NSW 1871 Australia

**Keywords:** Older refugee, Self-improvement, Resource, Aged care workers, Community service providers, Evaluation, Training

## Abstract

**Background:**

Resources and training for aged care workers who are working with older people from refugee backgrounds are limited. Thus, a resource titled ‘Enhancing the Lives of Older Refugees: A self-Improvement Resource for Community Service Providers’ was developed in 2011, and later accompanied by a training program developed in 2012. The aim of the resource and accompanying training was to assist community aged care service providers, based in South Western Sydney and surrounding areas, to recognise an older refugee, increase their knowledge and skills in working with older refugees, have a greater understanding of older refugees’ life experiences, and provide additional information that would allow them to offer appropriate services to those in their care. This paper reports on the evaluation of the training package provided to community aged care personnel.

**Methods:**

Eleven training sessions were conducted with all participants invited to take part in the research. One hundred and twenty-eight consenting participants completed a pre and post training evaluation questionnaire.

**Results:**

Analysis of the data indicated a positive change in participant’s ability to define an older refugee, understanding older refugee’s life experiences, loss and grief, the impact of the refugees’ experience in old age and the capacity to locate and access information to support the care of older refugees.

**Conclusions:**

The findings lend support that this mode of training can provide information and resources to increase the capacity of aged care workers to better meet the needs of older people from a refugee background.

**Electronic supplementary material:**

The online version of this article (doi:10.1186/s13033-016-0067-5) contains supplementary material, which is available to authorized users.

## Background

The plight of refugees is a growing international concern. The United Nations High Commissioner for Refugees (UNHCR) estimated that at the end of 2013 there were approximately 51.2 million people forcibly displaced by persecution and conflict [1]. Few countries worldwide participate in the UNHCR third country resettlement program, with Australia accepting approximately 13,750 refugees annually, making it the third leading resettlement nation [2].

A refugee is a person who has left their home country as a result of “a well-founded fear of persecution for reasons of race, religion, nationality, membership of a particular social group or political opinion” [3]. This persecution often involves serious human rights abuses, including torture, imprisonment and violence. Australia has resettled approximately 700,000 refugees since the end of World War II [2].

Evidence suggests that older refugees may have a difficult and differing resettlement process compared with younger refugees, “The age of a refugee on arrival in their country of resettlement will be crucial in determining their experience of resettlement” [4; p 16]. This is further supported by results of a systematic review [5] and other research studies [6, 7] which highlighted that older refugees have added challenges associated with the normal ageing process as well as feeling isolated and vulnerable in a foreign environment. Relatedly, in the report developed by the New South Wales Refugee Health Service titled “Caring for Older Refugees in NSW” [4] it was highlighted that many of the health problems and specific needs of Australia’s older refugees are not recognised by aged and health care services. Furthermore, it was noted that “Being able to identify whether a client has a refugee background requires skill” [4; p 43] and therefore resources to help services and staff identify if a client is from a refugee background were needed. According to the literature, older refugees face multiple challenges that are additional to those of the Australian-born and migrant elderly population, including higher risk of mental and physical health issues [8]. Refugees, by definition, have fled serious human rights violations because of war or organised violence. Some will be survivors of torture, some will have had children, siblings or parents who were killed, and all will have lost their homes, way of life and community [4]. These experiences can have a profound impact on their health, the way they will access services and the way aged care services need to be provided.

It is generally noted that there are two groups of older refugees living in NSW [9]. The first are those that have grown old in Australia after arriving many years ago as early as 1947. This group forms the largest in numbers and includes people who fled from Europe after WWII as well as Asia and South America in the 1970s and 1980s. The second group of older refugees are those who arrived as older persons as part of the Humanitarian Program or were reunited with their families through the broader migration program. It is this latter group that have high settlement needs [9]. Some will have escaped recent conflicts; others will be reuniting with family in Australia after many years of separation. Psychologically, it has been reported that older people from this latter group can feel that they are ageing “out of” or “in the wrong place” thereby increasing their levels of distress [9, 10].

Psychological health concerns of older refugees include isolation, depression and cognitive decline [11]. Disruptions to memory can trigger painful suppressed memories [11]. Evidence suggests that severe traumatisation can persist for many years after the actual trauma took place [12]. Further, it is argued that previous experiences may make it difficult to develop the trust needed to develop a supportive social network and confidence in dealing with mainstream organisations. Another factor that may act as a barrier for older people from culturally and linguistically diverse backgrounds (CALD) to access services is that they are often cared for in the home by their children, which is seen as a duty of care, responsibility and continuation of the family unit [13, 14]. As a result of these barriers, older refugees may only come to the attention of aged care services once they have very high complex needs and late presentations are common [4, 14]. This is supported by consumer and stakeholder feedback which indicates that many CALD older people, their families and carers tend to access aged care services when they reach a crisis rather than accessing basic services gradually [8].

Furthermore, despite research consensus supporting the notion that older refugees are a highly vulnerable group [8, 12, 15], indications are that aged care services are limited in caring for this group due to a low level of knowledge about how to identify and address their care needs [4, 14, 15]. In response to this need, ‘Enhancing the Lives of Older Refugees: A self-Improvement Resource for Community Service Providers’ was created, with input from the Older Refugee Working Committee (ORWC) [16], an extensive literature review, and community consultation. After development of the resource, the ORWC identified the need for training to support its use, as well as a need to evaluate the training. Specifically, training was provided to aged care service providers based in South Western Sydney and surrounding areas. As such the aim of this study was to evaluate the developed training resource and the accompanying training and to determine whether completion of such training improved the knowledge and capacity of community aged service providers in the provision of care for older refugees.

## Methods

The evaluation study was approved by the Western Sydney University Human Research Ethics Committee (H9985). Aged care managers and aged care community service provider coordinators were invited to participate in the older refugee training program. On registration, participants were informed that the training would be evaluated and were given an information pack consisting of a participant information letter about the evaluation process and a participant consent form. At the beginning of the training session, participants were again briefed on the study evaluation process, which consisted of a pre and post questionnaires. Participants were requested to complete and sign the research consent form along with the pre evaluation questionnaire if they chose to participate in the evaluation. One researcher provided the information to participants on the research project and another conducted the training. To ensure consistency the same trainer conducted all eleven training sessions. A set of post-evaluation questions was provided at the completion of the 2 h training session to consenting participants. The training consisted of a two-hour session covering the four key areas included in the resource. The key areas were older refugee’s individual life experience; impact of loss and grief on older refugee; older refugee ageing process and wider impact of the refugee experience on older age. The training was didactic however questions were encouraged from participants throughout the sessions. Examples from their everyday practices were used and facilitated by the trainer, linking them to the resource to encourage the on-going use of the self help resource once participants returned to their workplace.

The older refugee training program was conducted from July 2013 until June 2014. One hundred and twenty-eight participants (n = 128) consented to completing a pre and post evaluation questionnaire, which included collection of participant demographic details, five questions requiring likert-scale self-report responses for the pre evaluation designed to assess pre-existing knowledge, and nine likert-scale self-report responses for the post evaluation questions with additional multiple choice options assessing knowledge in response to a short case vignette which is presented in the appendix.

### Statistical analysis

Statistical analysis was carried out using IBM Statistics version 22.0 [17]. Data for continuous variables are presented as means and standard deviations (SD), and categorical variables are presented as frequencies and percentages. Changes in levels of agreement in participant’s knowledge and understanding pre and post training was elevated using Wilcoxon signed-rank test. Finally, the free text responses to a vignette were grouped into categories according to the frequency of responses.

## Results

### Participants

Table 1 presents the demographic characteristics of the research participants. Of the 81 (63.3 %) participants from overseas, 8 (6.3 %) reported having arrived as refugees, with the remaining participants arriving to Australia under other migration schemes. Majority of participants were female, reflective of the workforce in the aged care sector, which is predominantly female [18]. English was the most common language reported to have been spoken at home (48 %) followed by Polish with 18 %. Finally, 96 % of participants had no previous training related to older refugees.

**Table 1:** Demographic characteristics of participants

Characteristics	N (Total = 128)^a^	%
Gender		
Male	7	5.5
Female	121	94.5
Age categories		
18–35	18	14.1
36–45	23	18.0
46–55	47	36.7
56–65	34	26.6
66–75	2	1.6
Education categories		
High school	11	8.6
TAFE, Diploma’s or other certificates	48	37.5
Undergraduate university	49	38.3
Postgraduate university	16	12.5
Region of origin		
Australia	47	36.7
Europe	33	25.8
Asia	23	18.0
Middle east	9	7.0
Others	16	12.6

^a^May not add to 128 due to missing data

### Training evaluation

Table 2 presents the pre and post evaluation data from participants who attended training.

**Table 2:** Pre and post training evaluation

Concepts addressed in the training	Pre training, n (%)	Post training, n (%)
Define ‘Refugee’		
Strongly disagree	–	–
Disagree	3 (2.3)	–
Undecided	28 (21.9)	1 (0.8)
Agree	78 (60.9)	53 (41.4)
Strongly agree	15 (11.7)	71 (55.5)
Understand life experiences of the ‘Older Refugee’		
Strongly disagree	2 (1.6)	–
Disagree	14 (10.9)	2 (1.6)
Undecided	42 (32.8)	2 (1.6)
Agree	56 (43.8)	71 (55.5)
Strongly agree	11 (8.6)	52 (40.6)
Understand loss and grief experiences of the ‘Older Refugee’		
Strongly disagree	2 (1.6)	–
Disagree	13 (10.2)	–
Undecided	34 (26.6)	1 (0.8)
Agree	59 (46.1)	70 (54.7)
Strongly agree	18 (14.1)	56 (43.8)
Understand the impact of the ‘refugee’ experience on old age		
Strongly disagree	3 (2.3)	–
Disagree	13 (10.2)	1 (0.8)
Undecided	37 (28.9)	2 (1.6)
Agree	52 (40.6)	72 (56.3)
Strongly agree	21 (16.4)	52 (40.6)
Locate and access information for the care of ‘Older Refugees’		
Strongly disagree	3 (2.3)	–
Disagree	24 (18.8)	1 (0.8)
Undecided	44 (34.4)	6 (4.7)
Agree	51 (39.8)	85 (66.4)
Strongly agree	4 (3.1)	35 (27.3)

The pre and post evaluation survey contained five statements which reflected a core aspect or concept that was addressed in the training workshop and measured using a five-point likert rating scale ranging from ‘strongly disagree’ to ‘strongly agree’. In keeping with the resource structure, the five questions explored participant’s capacity to define an older refugee, their understanding of an older refugee’s life experiences, loss and grief, the impact of being an older refugee and finally the ability to locate and access resources to support their care of an older refugee when working in the community.

A series of Wilcoxon signed-rank tests demonstrated statistically significant increased learning and understanding across all five concepts following the training workshop. Specifically, there was significant increase in agreement by participants who reported they could now define the term “refugee” (Z = 7.647, *p* < *0.0001*) and were better able to understand the individual life experiences of an “older refugee” (Z = 7.842, *p* < *0.0001*). Further, participants were also better able to understand the key concepts related to being an older refugee such as issues around “loss and grief” (Z = 7.667, *p* < *0.0001*) and impact of the “refugee” experience on old age (Z = 7.114, *p* < *0.0001*). Finally, there was also significant change in agreement following training that participants could now better locate and access information to support their care of an older refugee (Z = 8.336, *p* < *0.0001*).

An additional four questions were also contained in the post training survey, which sought to address overall usefulness of the training, the training content, the influence of the training in assisting in care and the usefulness of the resource in assisting in the care of older refugees. Sixty percent ‘strongly agreed’ and 40 % ‘agreed’ that the training improved their ‘knowledge’ about older refugees. There were equal response ratings of ‘agreed’ and ‘strongly agree’ for the “training description accurately reflecting the content of the training” as was the “training assisting in care” and the “resource assisting in care” of the older refugee.

### Modified brief health literacy survey

In order to obtain a more objective measure of an increase in knowledge, participants were provided with a clinical vignette. The vignette described a fictional older Vietnamese refugee named ‘Anh’, who had been exposed to trauma in her homeland and was currently living alone after becoming a widow. She had come to the attention of her GP due to overall decline and increasing confusion. Following this vignette, two questions with six multiple-choice options and one open ended question were administered, designed to assess knowledge related to recognition of an older refugee and how to recognise grief and loss in an older refugee.

Eighty-one percent (n = 104) identified “country of birth”, 79 % (n = 101) identified “year of arrival” and 54 % (n = 69) identified “suspiciousness” as factors that would assist in determining whether a person meet the definition of an older refugee, which correctly reflected the training content. Seventy-four percent (n = 95) selected “preparing food for two or more people”, 85 % (n = 95) selected “reliving the past”, 79 % (n = 101) selected “open expressions of grief” and 54 % (n = 69) selected “visiting the temple” as common factors that could be expressed by an older refugee experiencing “loss and grief”. These were also the most correct responses. Overall, this method of assessment provided further support that the older refugee training program was effective in increasing participants’ knowledge to assist in the care of older refugees.

### Qualitative findings

An open ended question was provided to allow participants the option of identifying further information that may be required in planning care for the fictional character ‘Anh’. Individual participants’ statements, some of which are reported below, highlighted the importance of planning care that was person-centred and culturally specific.

- “Gather more information from her General Practitioner and her support networks. Find out about her coping abilities at this time.” (Participant 120, female, age rage 36–45).
- “In the assessment, listen to her, let her talk; find out what she would like her life to be..” (Participant 49, Female, age rage 46–55).
- “Help her to find her social environment that she will feel comfortable with. Some physical help if she needs it.” (Participant 119, Female, age rage 46–55).
- “Comprehensive assessment is required. Provide person-centred care and planning. Find out and build up her social inclusion.” (Participant 122, Female, age rage 46–55).
- “Connect the consumer with her community. Consult post trauma experts. Educate self and the other workers involved.” (Participant 124, Female, age rage 46–55).
- “Social support/social companion needed. Organise a staff member with a similar refugee and language background.” (Participant 12, Female, age rage 26–35).
- “Organise language support by using an Interpreter and organising a bilingual worker.” (Participant 45, Female, age rage 46–55).
- “Engage the consumer and identify what she would like to achieve, work on her goals.” (Participant 35, Male age rage 56–65).

## Discussion

This study aimed to evaluate the ability of the older refugee training program, consisting of a written resource titled ‘Enhancing the Lives of Older Refugees: A self-Improvement Resource for Community Service Providers’ and the accompanying training, to improve the care of older refugees. This training package sought to address the dearth of resources and training available for community aged care workers working with older refugees. Results of the training indicated a positive change in participants’ ability to recognise who is an older refugee, understand important concepts related to the care of an older refugee and locate resources to aid in the care of an older refugee. Furthermore, this positive change was confirmed when participants’ knowledge was examined using a more objective assessment in the form of a health literacy vignette. Overall, our findings confirmed the successful impact of our training resource in helping to improve the capacity of aged care workers to better meet the needs of older people from a refugee background.

Further evaluation of the training, which included responses to open-ended questions, highlighted the need for comprehensive assessment including listening to the client, engaging the General Practitioner, identifying the client’s goals and coping abilities and using an interpreter to enhance communication. The need for a person centred approached to planning care, was frequently noted by participants. These findings are consistent with previous research [7] that highlighted the importance for aged care workers to understand and take into consideration each client’s own life history that is relevant to their care. Workers need to recognise older refugees physical and emotional needs, have connection with the client’s community and links to specialist post trauma experts. Moreover participants referred to the need for the client to be socially engaged, provided with social support and companionship and to be linked with a bilingual support worker.

Another interesting finding that emerged from our study was the fact that despite 81 % of the participants identifying as being born overseas, of which 52 % were from a non-English speaking background, a number of these participants stated in general feedback that they were unaware that some of the people from countries mentioned in the training manual could be from a refugee background [16]. This may indicate that the assumption that people from CALD background have a greater recognition of an older refugee and their life experiences compare to those born in Australian is incorrect. This is consistent with research on the mental health literacy of clinical populations which has noted that recognition of mental health disorders, including self-recognition can be poor and is a function of a number of factors such as age, sex and education levels [19]. This further strengthens the argument that all age care workers would benefit from training such as ours.

## Limitations

The study had a number of limitations. This included the reduced capacity to roll out the training beyond the initial year due to funding considerations. Another was due to volunteer nature of training participation; representativeness of aged care workers cannot be ascertained. Finally, while the evaluation of our training demonstrated immediate increase in participant’s knowledge, the ability to assess the retaining of this knowledge over time would have been useful if funding permitted a follow-up arm. Strengths of the study include the delivery of the training program by one investigator thus ensuring consistency and training fidelity. Additionally, the assessment of the increase in participants knowledge utilising multiple methods of self-report and health literacy added rigour to the evaluation of the study. Finally, of greatest testament to the acceptability and merit of our older refugee training program were the consistent requests from aged care service providers to deliver the training program to other workers in their team.

## Conclusion

The study sought to evaluate a training resource developed specifically for aged care service providers. Study findings indicated that the training had a positive influence on participant’s ability to define an older refugee, understand older refugee’s life experiences such as loss and grief, the impact of the refugees’ experience in old age and the capacity to locate and access information to support the care of older refugees. Findings confirm that this mode of training can provide information and resources to increase the capacity of aged care worker to better meet the needs of older people from a refugee background (Additional file 1).

## Additional file

10.1186/s13033-016-0067-5 The vignette used in the health literacy survey.

## Abbreviations

UNHCR: United Nations High Commissioner for Refugees
CALD: culturally and linguistically diverse backgrounds
ORWC: Older Refugee Working Committee
