# Supplementary material for: Enhancing the lives of older refugees: an evaluation of a training resource
Source: Int J Ment Health Syst. 2016 Apr 29;10:36. doi: 10.1186/s13033-016-0067-5 (PMC4850683; doi:10.1186/s13033-016-0067-5)
Supplement: Supplementary file 1 — 10.1186/s13033-016-0067-5 The vignette used in the health literacy survey. [file 13033_2016_67_MOESM1_ESM.docx]

Additional file 1 – **The vignette used in the health literacy survey**

Anh is a 74-year-old female, who arrived from Vietnam in 1980. She lives alone after her husband died. Her Vietnamese speaking GP was concerned about her ability to cope, with a change in her presentation and level of social engagement. He referred her to Neighbour Aid for support from a Vietnamese volunteer. A good relationship was fostered including visiting the temple regularly. Anh shared life stories telling about the trauma she experienced in Vietnam and of her journey to find freedom. Her only son died at sea on the way to a refugee camp in Malaysia. The volunteer noted Anh’s gradual decline and confusion. She seemed to be cooking a lot of food for just one person and kept talking about her son and husband with tears. She is also becoming highly suspicious and disoriented. This was reported to the service manager who re-referred her to the GP
